# Radiomics models based on CT at different phases predicting lymph node metastasis of esophageal squamous cell carcinoma (GASTO-1089)

**DOI:** 10.3389/fonc.2022.988859

**Published:** 2022-10-26

**Authors:** Guobo Peng, Yizhou Zhan, Yanxuan Wu, Chengbing Zeng, Siyan Wang, Longjia Guo, Weitong Liu, Limei Luo, Ruoheng Wang, Kang Huang, Baotian Huang, Jianzhou Chen, Chuangzhen Chen

**Affiliations:** ^1^ Department of Radiation Oncology, Cancer Hospital of Shantou University Medical College, Shantou, China; ^2^ Department of Radiation Oncology, Meizhou People’s Hospital (Huangtang Hospital), Meizhou Academy of Medical Sciences, Meizhou, China; ^3^ Shantou University Medical College, Shantou, China

**Keywords:** esophageal squamous cell carcinomas, esophageal cancer, lymph node metastasis, radiomics, computed tomography, prediction model

## Abstract

**Purpose:**

To investigate the value of radiomics models based on CT at different phases (non-contrast-enhanced and contrast-enhanced images) in predicting lymph node (LN) metastasis in esophageal squamous cell carcinoma (ESCC).

**Methods and materials:**

Two hundred and seventy-four eligible patients with ESCC were divided into a training set (n =193) and a validation set (n =81). The least absolute shrinkage and selection operator algorithm (LASSO) was used to select radiomics features. The predictive models were constructed with radiomics features and clinical factors through multivariate logistic regression analysis. The predictive performance and clinical application value of the models were evaluated by area under receiver operating characteristic curve (AUC) and decision curve analysis (DCA). The Delong Test was used to evaluate the differences in AUC among models.

**Results:**

Sixteen and eighteen features were respectively selected from non-contrast-enhanced CT (NECT) and contrast-enhanced CT (CECT) images. The model established using only clinical factors (Model 1) has an AUC value of 0.655 (95%CI 0.552-0.759) with a sensitivity of 0.585, a specificity of 0.725 and an accuracy of 0.654. The models contained clinical factors with radiomics features of NECT or/and CECT (Model 2,3,4) have significantly improved prediction performance. The values of AUC of Model 2,3,4 were 0.766, 0.811 and 0.809, respectively. It also achieved a great AUC of 0.800 in the model built with only radiomics features derived from NECT and CECT (Model 5). DCA suggested the potential clinical benefit of model prediction of LN metastasis of ESCC. A comparison of the receiver operating characteristic (ROC) curves using the Delong test indicated that Models 2, 3, 4, and 5 were superior to Model 1(P< 0.05), and no difference was found among Model 2, 3, 4 and Model 5(P > 0.05).

**Conclusion:**

Radiomics models based on CT at different phases could accurately predict the lymph node metastasis in patients with ESCC, and their predictive efficiency was better than the clinical model based on tumor size criteria. NECT–based radiomics model could be a reasonable option for ESCC patients due to its lower price and availability for renal failure or allergic patients.

## Introduction

Esophageal cancer is one of the most common malignancies affecting people’s health, which ranked seventh in morbidity (604,000 new cases) and sixth in overall mortality (544,000 deaths) worldwide in 2020 (1). The 5-year survival rate is only 20% to 30% (2). The subtypes of esophageal cancer are mainly composed of esophageal squamous cell carcinoma (ESCC) and adenocarcinoma. In China, esophageal squamous cell carcinoma accounts for about 90% of esophageal cancers (2, 3).

Most patients with esophageal cancer require comprehensive treatment, with surgery or endoscopic resection as the main treatment in the early stage, and concurrent radiotherapy and chemotherapy as the first choice for patients in the middle to advanced stage (4). The status of lymph node metastasis not only has an important influence on the choice of therapeutic regimen for EC, but also is a superior prognostic indicator (5, 6). However, the existing inspection instruments have their shortcomings in the estimation of lymph node metastasis.

Endoscopic ultrasonography, as an invasive examination, has a sensitivity of 80%, but it is highly dependent on the operator, and about 30% of patients cannot complete this examination because of esophageal stenosis (3, 7). Magnetic resonance imaging (MRI) has a high contrast resolution in soft tissue imaging, but it is very time-consuming and costly. PET/CT is a rapidly developed new imaging equipment that organically combines positron emission tomography with X-ray tomography. However, due to its high price, low sensitivity, and high false positive rate, its application in the diagnosis of lymph node metastasis is limited (7, 8). Computed tomography (CT) is a common imaging technique for the diagnosis of metastatic lymph nodes but it is mainly based on morphological criteria by measuring the maximum short diameter of the lymph node, with an unsatisfactory accuracy and sensitivity of less than 60% (9). How can we use non-invasive diagnostic tools such as medical imaging to further improve the accuracy of predicting lymph node metastatic status of esophageal cancer? The solution to this problem is of great clinical significance for the management of esophageal cancer patients and precision medicine.

In recent years, the development of radiomics provides an opportunity for noninvasive prediction of lymph node metastatic status. The concept of radiomics was first proposed by Lambin et al. in 2012. It is defined as the extraction and analysis of a large number of advanced quantitative imaging features from images with high throughput (10, 11). Quantitatively analyzing these data can assist in clinical diagnosis and the treatment of tumors.

At present, radiomics has achieved much remarkable progress in the qualitative and prognostic prediction of tumors, including lung cancer, breast cancer and colorectal cancer (12–14). Previous studies have suggested that objective and quantitative imaging features obtained from CT may be used as predictive biomarkers. It has much less invasiveness compared to biopsy or other similar procedures. To our knowledge, most previous studies have just focused on images of a single phase, such as contrast-enhanced period, which may not maximize the potential value of different phases of CT image (15, 16). In our study, for the first time, both NECT and CECT were used for quantitative analysis to predict lymph node metastasis of ESCC.

## Methods and materials

### Study population

This retrospective study of anonymous data involving human participants was reviewed and approved by the Ethics Committee of Cancer Hospital of Shantou University Medical College (No.2021121). The written informed consent was not required for this study. Two hundred and seventy-four consecutive patients with ESCC, who were divided into the training group (n=193) and the validation group (n=81) in Cancer Hospital of Shantou University Medical College from October 2016 to September 2018 were enrolled in this study, according to the following criteria:

Inclusion criteria: 1) patients who underwent radical resection of esophageal carcinoma which were pathologically confirmed to be ESCC afterwards; 2) patients with a complete record of lymph node metastasis in the postoperative pathological report; 3) patients who received NECT and CECT scan within 1 week before surgery.

**Figure 1:**
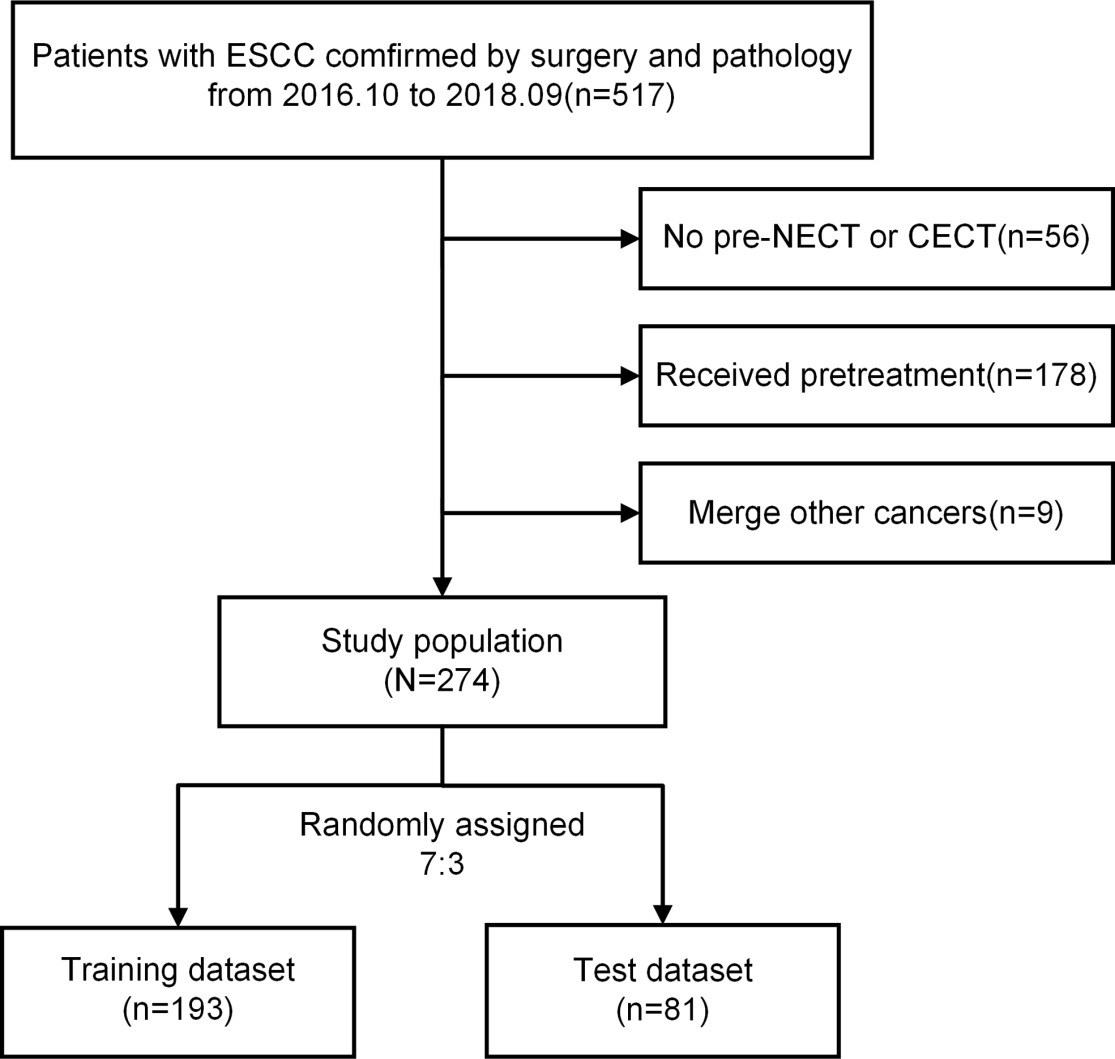
Flowchart of the exclusion and inclusion processes. ESCC, esophageal squamous cell carcinoma; NECT, non-contrast-enhanced computed tomography; CECT, contrast-enhanced computed tomography.

Exclusion criteria: 1) patients who received preoperative neoadjuvant therapy, such as radiotherapy, chemotherapy, immunotherapy and so on; 2) patients who lacked CT images or had low-quality image that cannot be evaluated; **Figure 1** shows the flowchart of case inclusion and exclusion.

Clinical data including demographic data (age, gender), tumor location, CT image report, lymph node metastasis status was collected from the hospital information system. In addition, neutrophil lymphocytes ratio, platelet lymphocytes ratio, hemoglobin and other values were collected before treatment (17).

When CT images were analyzed, lymph node metastatic status was considered positive as the maximum short diameter of the lymph node > 1cm or inhomogeneous enhancement of the lymph node (CT-reported LN positive). The location of esophageal tumors was determined according to the 8th edition of the American Joint Committee on Cancer (AJCC) Cancer Staging Manual. N0 was defined as negative lymph node metastasis and N1-3 was defined as positive lymph node metastasis (18).

### Image acquisition and normalization

Prior to treatment within a week, all patients underwent CT scans from the neck to the upper abdomen in head first supine position (CT scanner: 16-row Spiral CT of Bright Speed ​​Series of GE Medical Systems, USA). CT scanning parameters were setting as follows: Tube voltage,120KpV; Rotation time, 0.75 seconds; Pitch, 1.375; Matrix, 512×512; Field of visual, 360 mm×360 mm. First, plain CT scan was performed to collect NECT images. Then, 70-80ml of iodine contrast agent was injected into cubital vein with the rate of 2.0ml/s, and CECT images were collected 22-25 seconds later. After the completion of scanning, the original data was transferred to GE workstation for 3D reconstruction with the slice thickness as 5mm. Image normalization for sharper differences between dark and bright regions was implemented in Python.

### Radiomics workflow

#### Tumor segmentation and feature extraction

NECT and CECT images (DICOM format) of all patients in this cohort were imported into 3D-slicer (an open source software: https://www.slicer.org/).

Lymph nodes larger than 1cm are commonly considered as metastatic in clinical. However, some studies has showed that lymph node size is not a reliable parameter for the evaluation of lymph node metastasis (19, 20). In some previously published literature, esophageal tumors were used to delineate regions of interest (15, 16). Therefore, the esophageal tumor area was defined as the region of interest (ROI) in this study. A radiotherapist with more than 10 years of experience performed manual segmentation to delineate the gross tumor volume of ESCC layer by layer on CECT images on the axial view, and avoided the interference of blood vessels, fat and bone to obtain the ROI. Esophageal wall thickness greater than 5 mm on the transverse axis of CT image is considered abnormal.

The ROI obtained from the CECT image was compared with the tumor area delineated on the NECT image and adjusted appropriately. And then another radiotherapist with 15 years of experience randomly selected 30 of the CT images and performed ROI segmentation again. Intraclass correlation coefficient (ICC) was used to evaluate the inter-observer reliability (21). ICC greater than 0.75 was considered to have good repeatability or stability in radiomics.

The radiomics features of each patient were extracted from ROI using the pyradiomics v3.0 package in Python v3.7.6 software. (https://pyradiomics.readthedocs.io/en/latest/index.html)

The extracted features are subdivided into eight categories:

I, First Order Statistics;
II, Shape-based Features (3D/2D);
III, Gray Level Cooccurrence Matrix (GLCM);
IV, Gray Level Size Zone Matrix (GLSZM);
V, Gray Level Run Length Matrix (GLRLM);
VI, Neighboring Gray Tone Difference Matrix (NGTDM);
VII, Gray Level Dependence Matrix (GLDM);
VIII, High Older Features.

**Figure 2:**
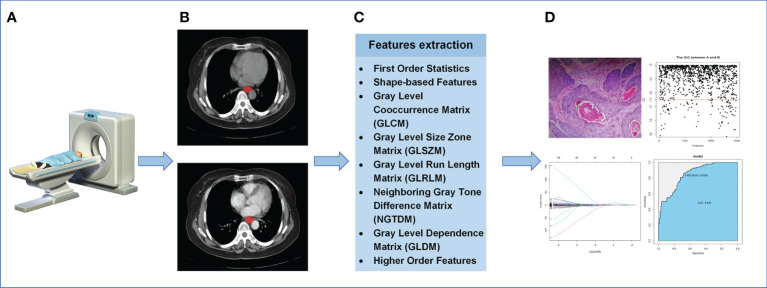
The process of radiomics. **(A)** Acquisition of CT images. **(B)** Segmentation of ROI. **(C)** Extraction of ROI feature. **(D)** Statistical analysis.

In addition, when extracting higher-order features, the Sigma parameter was set to 0.5, 1.0, 1.5, 2.0, and five image types were added derived from the original image features(LoG, Wavelet, Square, SquareRoot, Logarithm).15 filters were applied to the original image to obtain derivative image of each patient. The extracted radiomics features will be used for subsequent statistical analysis. The process of radiomics was shown in **Figure 2**. The endpoint of this study was the pathological diagnosis of lymph nodes. More information about the radiomics features was applied in appendix.

#### Feature selection and model establishment

Feature selection included four steps. Firstly, ICC was used to estimate the reproducibility of each radiomics feature. In our study, CECT features with ICC less than 0.75 were excluded. And the same features in NECT were excluded as well. Secondly, in order to eliminate the impact of dimensional and value range differences, Z-score was adopted to standardize the features with good stability screened out by ICC. Thirdly, tests of normality and homogeneity of variance were applied to the remaining features. The independent sample T test was used for the features that follow normal distribution and had homogeneous variance, and the Mann-Whitney U test was used for the rest to further eliminate the non-significant features (P > 0.05). Finally, to minimize the overfitting in the study, the LASSO was performed to eliminate reductant features on the training group (22). Ten-folder cross validation was performed on the training dataset to produce the predictive models. The features in those models were preserved as optimal features of nonzero coefficients on NECT and CECT images, respectively. The variance inflation factor (VIF) was used to evaluate the collinearity of retained features (23). VIF value greater than 10 is generally considered that there is serious multicollinearity among variables.

**Figure 3:**
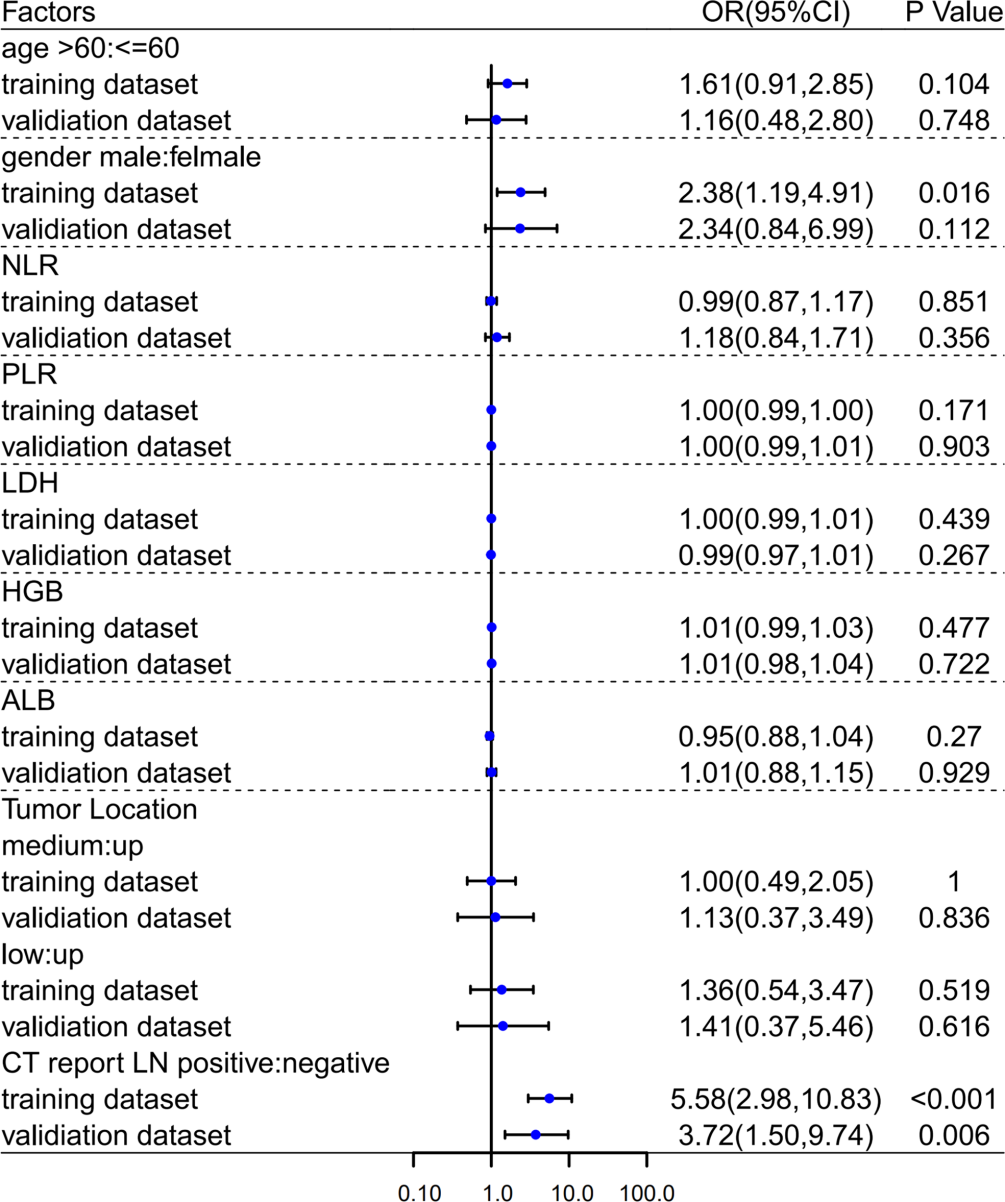
Univariate analyses in training and validation dataset. OR, Odds Ratio; 95%CI, 95% confidence interval.

Through the above screening steps, the most significant features were used to establish the radiomics signatures of NECT and CECT images. Univariate analysis was used to compare clinical factors which were listed in **Figure 3** between pathologically confirmed LN positive patients and LN negative patients. Significant different factors were then analyzed by multivariate analysis to determine clinical factors included in our models.

Combined with clinical factors and radiomics signatures, five models were developed to predict lymph node metastatic status as follows: Model 1 (only clinical factors), Model 2 (clinical factors & Radiomics_NECT_), Model 3 (clinical factors & Radiomics_CECT_), Model 4 (clinical factors & Radiomics_NECT_ & Radiomics_CECT_), Model 5 (Radiomics_NECT_ & Radiomics_CECT_). The model output was defined as the systematic pathological description of status of lymph nodes.

The performances of the models were evaluated by area under receiver operating characteristic curve (AUC), sensitivity, specificity and accuracy. The calibration curve was used to evaluate the consistency of the models’ prediction results with actual LN metastasis of ESCC. DCA was used to evaluate the models’ clinical application value. DeLong test was used to compare the AUC between models (24).

### Statistical analysis

All statistical analysis was performed in R (version 4.01; https://www.r-project.org/). The “psych” package was used to calculate the ICC. The “glmnet” package was used for LASSO analysis and feature reduction. The “pROC” package was used to calculate the AUC and draw the ROC curves. The glm () function in the “RMS” package was used for logistics regression analysis to determine the clinical risk factors related to lymph node metastasis of ESCC. The “rmda” package was used to draw the clinical decision curves.

For the differences in clinical characteristics between the training group and the validation group, independent sample T test and Mann-Whitney U test were used for continuous variables, and chi-square test was used for categorical variables such as gender and tumor location. P value less than 0.05 on both sides indicated a statistically significant difference.

## Result

### Patients’ characteristics

A total of 274 patients who met the criteria were randomly assigned in a 7:3 ratio to the training group (n=193) and the test group (n=81). A detailed comparison of the characteristics of the training and test groups was shown in **Table 1**.

**Table 1:** Baseline characteristics of 274 ESCC patients in the training and validation dataset.

Variable	Training dataset (n=193)	Validation dataset (n=81)	p-value
Age (years)			0.880^a^
<=60	87 (45.08%)	35 (43.21%)	
>60	106 (54.92%)	46 (56.79%)	
Gender			0.783^a^
Female	43 (22.28%)	20 (24.69%)	
Male	150 (77.72%)	61 (75.31%)	
Tumor location			0.583^a^
Up	40 (20.73%)	17 (20.99%)	
Medium	120 (62.18%)	46 (56.79%)	
Low	33 (17.1%)	18 (22.22%)	
CT-report LN status			0.999^a^
Negative	109 (56.48%)	46 (56.79%)	
Positive	84 (43.52%)	35 (43.21%)	
Pathological LN status			1.000^a^
Negative	94 (48.7%)	40 (49.38%)	
Positive	99 (51.3%)	41 (50.62%)	
NLR Median	2.35	2.29	0.252^b^
IQR	1.71 to 3.21	1.80 to 3.28	
PLR Median	130.72	132.35	0.985^b^
IQR	93.12 to 174.17	106.26 to 176.09	
LDH (U/L) Mean ± sd	161.62 ± 27.09	167.54 ± 26.88	0.099^b^
HGB (g/L) Mean ± sd	136.50 ± 15.09	136.46 ± 15.13	0.980^b^
ALB (g/L) Mean ± sd	42.75 ± 3.51	42.51 ± 3.23	0.580^b^

ESCC, esophageal squamous cell carcinoma; LN, lymph node; NLR, neutrophil lymphocytes ratio; PLR, platelet lymphocytes ratio; LDH, lactate dehydrogenase; HGB, hemoglobin; ALB, albumin.

a p-value was calculated using chi-square test;

b p-value was calculated using independent sample T test or Mann-Whitney U test;

The pathologically positive lymph node metastasis rates of the training group and the validation group were 51.3% and 51.62%, respectively. There were no statistically significant differences in clinical and pathological features between the two datasets, with P-values ranging from 0.099 to 1.000.

In both univariate (**Figure 3**) and multivariate (**Table 2**) analyses, CT image reported lymph node metastatic status (i.e., morphological criteria) was a clinical risk factor for pathologically mediastinal lymph node metastasis of ESCC in both two datasets.

**Table 2:** Multivariate analysis in training and validation dataset.

Variables	Training dataset				Validation dataset		
	Coef	OR (95%CI)		P	Coef	OR (95%CI)	P
Intercept	-1.156	0.315		0.001	-1.116	0.328	0.035
		(0.150-0.624)				(0.107-0.885)	
Gender	0.753	2.123		0.485	0.785	2.193	0.161
		(1.016-4.572)				(0.746-6.884)	
CT-report LN status	1.459	4.303		<0.001	1.284	3.611	0.008
		(2.338-8.116)				(1.434-9.559)	

Coef, Coefficient.

### Feature selection

**Figure 4:**
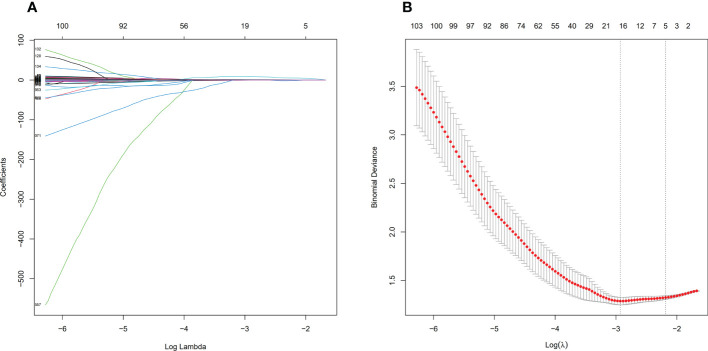
Feature selection process by LASSO in NECT. **(A)** shows the shrinkage diagram of 1216 radiomics features’ coefficients from NECT. **(B)** shows the 10-fold cross-validation curve, 16 optimal features were chosen based on the lambda.min criteria. LASSO, least absolute shrinkage and selection operator.

**Figure 5:**
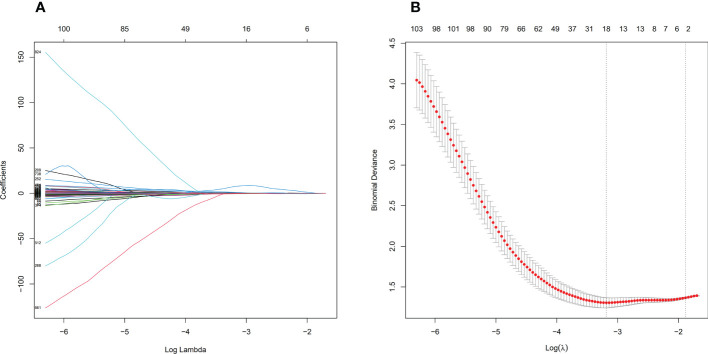
Feature selection process by LASSO in CECT. **(A)** shows the shrinkage diagram of 1227 radiomics features’ coefficients from CECT. **(B)** shows the 10-fold cross-validation curve, 18 optimal features were chosen based on the lambda.min criteria. LASSO, least absolute shrinkage and selection operator.

Before feature selection, we extracted 1502 radiomics features from NECT and CECT images respectively, which could be divided into 8 categories, including 18 histogram features, 14 morphological features, 75 original texture features and 1395 high-order features. Based on ICC > 0.75 standard, 1335 radiomics features were retained and 166 features were discarded because of large interobserver differences. 119 features from NECT images and 108 features from CECT images were discarded through the independent sample t-test or Mann-Whitney U test, respectively (P > 0.05). Finally, through LASSO regression algorithm, 16 and 18 non-zero coefficient features were selected from NECT images (**Figure 4**) and CECT images (**Figure 5**) respectively.

The VIF of 16 predictors from NECT images ranged from 1.13 to 7.14, and the VIF of 18 predictors from CECT images ranged from 1.15 to 6.89, indicating that there was no collinearity among the features. Specific filtered features and weight coefficients were applied in the supplementary material.

Based on the LASSO regression algorithm, the radiomics signatures of NECT and CECT were established. The specific formula was shown as follows:


Radiomics signature=Intercept_+ coef_1×feature_1+coef_2×feature_2+coef_3×feature_3+coef_4×feature_4+coef_5×feature_5+…+coef_n×feature_n(coef represents for coefficient)

### Model establishment and evaluation

**Figure 6:**
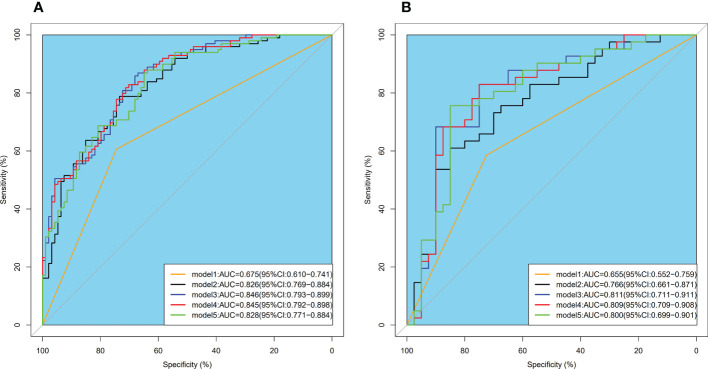
ROC curves of model 1-5 intraining **(A)** and validation **(B)** dataset. ROC, receiver operator characteristic; AUC, area under the curve.

**Figure 7:**
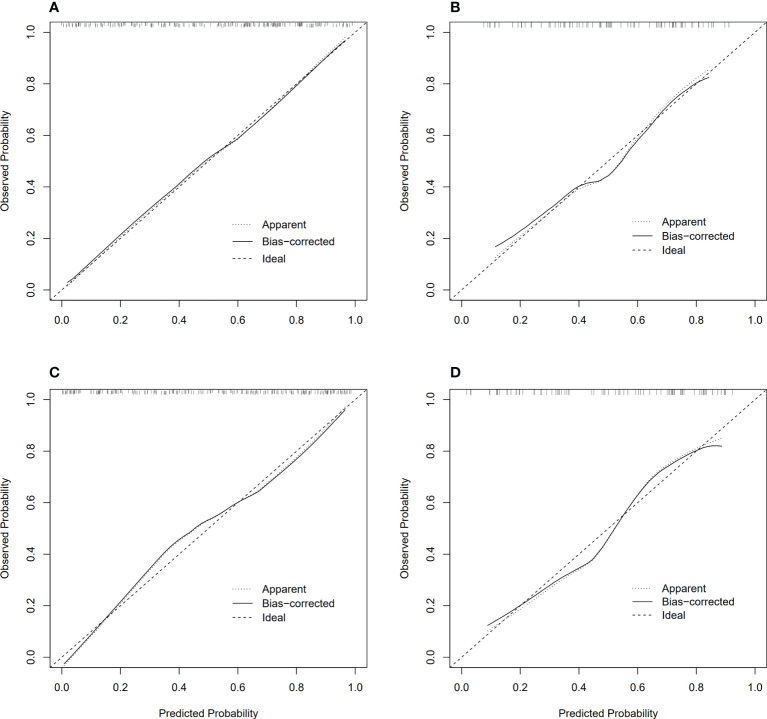
Calibration curves of model 2, 3 in training and validation dataset, respectively. **(A)** Calibration curves of model 2 in training dataset; **(B)** Calibration curves of model 2 in validation dataset; **(C)** Calibration curves of model 3 in training dataset; **(D)** Calibration curves of model 3 in validation dataset.

Model 2 reached an AUC = 0.826 in the training dataset and AUC = 0.766 in the validation dataset. Model 3-5 reached an AUC > 0.8 in both training and validation dataset, which indicate a considerable predictive value of LN metastasis. The detailed predictive performances of the Model 1-5 were presented in **Table 3**. The ROC curves of model 1-5 in training and validation dataset were shown in **Figure 6**. The calibration curves showed that Model 2,3 are in good agreement with actual observations, both in the training and validation dataset (**Figure 7**).

**Table 3:** ROC curves of the training dataset and the validation dataset.

model	group	AUC (95%CI)	sensitivity	specificity	accuracy
Model 1	Training	0.675 (0.610-0.741)	0.606	0.745	0.674
	Validation	0.655 (0.552-0.759)	0.585	0.725	0.654
Model 2	Training	0.826 (0.769-0.884)	0.789	0.734	0.762
	Validation	0.766 (0.661-0.871)	0.609	0.850	0.728
Model 3	Training	0.846 (0.793-0.899)	0.859	0.681	0.772
	Validation	0.811 (0.711-0.911)	0.683	0.900	0.790
Model 4	Training	0.845 (0.792-0.898)	0.818	0.713	0.767
	Validation	0.809 (0.709-0.908)	0.829	0.750	0.790
Model 5	Training	0.828 (0.771-0.884)	0.869	0.649	0.762
	Validation	0.800 (0.699-0.901)	0.756	0.850	0.802

ROC, receiver operator characteristic; AUC, area under the curve.

**Figure 8:**
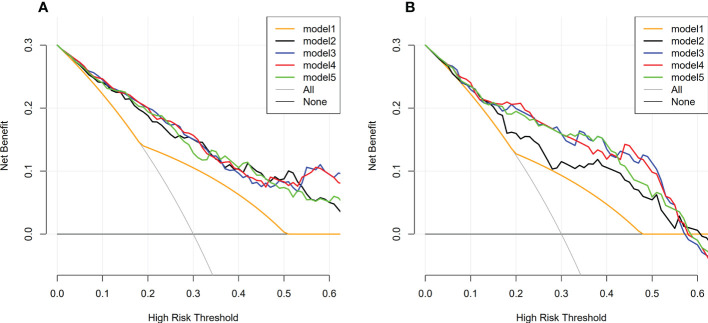
Decision curve analysis of model 1-5 in training and validation dataset. **(A)** Decision curve analysis of model 1-5 in training dataset; **(B)** Decision curve analysis of model 1-5 in validation dataset; The y-axis represents the net benefit. The x-axis represents the threshold probability. Model 2-5 had higher net benefits than Model 1, within a range of reasonable threshold probability.

The decision curve analysis showed that in most cases, compared with other therapeutic strategies (therapy for all patients, all patients without therapy, treat according to size criteria), Model 2-5 had higher net benefits than Model 1, within a range of reasonable threshold probability (**Figure 8**).

As shown in **Figure 6**, the AUC of Model 2-4 with the addition of radiomics features showed remarkable performance compared with Model 1. The result of Delong Test suggested that there was a statistical difference in the comparison between Model 1 and Model 2-5 (P<0.05). Among Model 2-5, Model 3 yielded the highest AUC of 0.846 in training dataset and 0.811 in validation dataset, but there was no statistical difference among Model 2-5. Details of the pairwise comparisons between the models were shown in the **Table 4**.

**Table 4:** Comparison among ROC curves of model 1-5 in training and validation dataset by Delong Test.

Models	Training dataset	Validation dataset
Model 1 vs Model 2	P<0.001	P=0.025
Model 2 vs Model 3	P=0.239	P=0.108
Model 2 vs Model 4	P=0.200	P=0.082
Model 2 vs Model 5	P=0.954	P=0.192
Model 3 vs Model 4	P=0.709	P=0.661
Model 3 vs Model 5	P=0.175	P=0.573
Model 4 vs Model 5	P=0.180	P=0.614

ROC, receiver operator characteristic.

## Discussion

Lymph node metastasis status is considered to be an important risk factor affecting the prognosis of patients, which is related to the scope of lymph node dissection during the operation, the need for neoadjuvant chemoradiotherapy, and the scope of radiotherapy for patients who are not suitable for surgery, or who cannot tolerate chemotherapy (5, 25, 26). The status of lymph node also has an important influence on the choice of therapeutic regimen for EC because whether neoadjuvant chemoradiotherapy or chemotherapy is needed before surgery mainly depends on the lymph node status. Since neoadjuvant chemoradiotherapy targets micro-metastases (including lymph node metastases), patients with lymph node metastases are likely to benefit from this therapy. For patients who refuse or cannot tolerate surgery, the status of lymph nodes cannot be diagnosed from postoperative biopsy. Definitive chemoradiotherapy is a common therapeutic regimen and the status of lymph nodes is also important for this kind of patients. Therefore, the preoperative prediction of lymph node status is necessary and important. However, there is currently no convenient or accurate tool for predicting lymph node metastasis status before treatment.

At present, CT is the conventional tool for clinical diagnosis of ESCC, but its prediction of lymph node metastasis status is based on morphological criteria, which may be biased due to inter-observer and intra-observer variability and affect the accuracy of diagnosis and staging. There were no unified criteria for CT diagnosis of lymph node metastasis nowadays. Lymph nodes smaller than 1 cm in short-axis diameter and have a smooth well-defined border, uniform homogeneous attenuation, and a central fatty hilum in CT are usually considered normal. Hong SJ etc. expounded that detection of lymph nodes metastatic state at CT imaging primarily depends on size criteria. Intrathoracic and abdominal lymph nodes larger than 1 cm in short-axis diameter and supraclavicular lymph nodes larger than 5 mm in short-axis diameter are considered to be metastatic lymph nodes. However, for normal-sized lymph nodes that contain microscopic metastatic foci, it is difficult to differentiate from non-metastatic lymph nodes at CT image, which can lead to understaging. On the contrary, benign, enlarged, inflammatory lymph nodes at CT imaging may lead to overstaging. In addition, metastatic lymph nodes close to esophageal cancer may not be detected because they are difficult to separate from the primary tumor. The number of lymph node metastases cannot be accurately measured if there are conglomerated lymph nodes which makes the N category difficult to be determined (27). Previous studies (7, 9) have shown that morphological criteria (mostly based on lymph node size) cannot effectively discriminate the lymph node metastatic status of ESCC, with a sensitivity less than 60%. In our study, the AUC of the model built by morphological criteria was 0.675 and 0.655 in training and validation dataset, respectively, which was basically consistent with the literature reports (15, 16).Previous studies have strongly demonstrated the feasibility of radiomics in bladder, colorectal, and breast cancer (12–14, 28). Studies had demonstrated that models integrating with radiomics features outperformed size criteria in discriminating lymph node metastasis in ESCC. Shen et al., Tan et al., developed a radiomics nomogram based on pre-treatment CECT images for the prediction of lymph node metastasis status in ESCC, and the AUCs of the training cohort were 0.806 and 0.758, respectively, and 0.771 and 0.773, respectively, in the validation cohort (15, 16). In a study of 411 CECT images from two medical centers, Wu et al. showed that adding computer vision and deep learning features on the basis of handcraft feature signatures could improve the model’s prediction value of lymph node metastasis in patients with ESCC (29). The above studies had focused on CT images of a single phase, which may not be able to find the best model from a series of CT images.

In this study, we extracted the quantitative image features of tumor from both NECT and CECT images. Sixteen and eighteen optimal radiomics features were selected from NECT and CECT images, respectively. The high-order texture features, including busyness and entropy were derived from NGTDM and GLRLM. Our study showed that higher value of entropy and busyness might all be associated with poorer overall survival. Entropy is a feature parameter to measure the randomness of image gray distribution, which represents the complexity of image texture. The more complex the image texture, the higher the entropy value (30). Busyness is used to describe the visual properties of a texture, based on the grayscale difference between a pixel and its neighbors. Studies have shown that high entropy is associated with the malignancy of lung cancer, liver cirrhosis and adnexal tumors (31–34). Fujimoto et al. reported that combined assessment of mean ADC and entropy ADC in patients with chronic hepatitis C more accurately predicted pathological liver fibrosis stage and inflammatory activity grade than assessment of mean ADC alone, helping to find early fibrosis or inflammatory activity. To more intuitively relate multiple features to the pathophysiological basis of tumors, we constructed multi-feature signatures that provide novel oncology biomarkers for obtaining phenotypic information, potentially helping clinicians develop management strategies.

A total of five logistic regression models were established: Model 1 (only clinical factors), Model 2 (clinical factors & Radiomics_NECT_), Model 3 (clinical factors & Radiomics_CECT_), Model 4 (clinical factors & Radiomics_NECT_ & Radiomics_CECT_), Model 5 (Radiomics_NECT_ & Radiomics_CECT_). The results show that models comprised of radiomics features had better performance than physicians in determining lymph node metastasis status based on CT morphology, and there was statistical difference between Model 1 and Model 2-5 calculated by Delong test (all P< 0.05). For example, between Model 1 and Model 2, we found significant improvements in the net reclassification index (NRI) of the model after adding the radiomics signature of NECT on the basis of clinical factors (NRI, 17.11% in training dataset, and 14.94%, in the validation dataset, respectively). Although the AUC of Model 2 in both the training dataset and the validation dataset (0.826 and 0.766, respectively) seemed lower than the others among models 2-5, the difference among them was not statistically significant (all P value > 0.05), which meant that they had the same predictive performance. It also supported the conclusion that the model combining clinical factors and radiomics_CECT_ could be an effective tool for predicting lymph node metastasis of ESCC before treatment, which was consistent with previous findings (15, 16).

Previous study has showed that compare to enhanced CT, the texture parameters extracted from plain CT images have more parameters with statistically significant difference (35). However, their study did not use Delong test to compare the models.In our study, we found that there was no statistical difference between the combined models of NECT and CECT after calculation using Delong test. Our study highly suggested that the NECT images also contained abundant tumor biological information. Compared to models comprised of radiomics_CECT_, the model combining clinical factors and radiomics_NECT_ had the same predictive power, which was good news for patients who do not have access to enhanced CT scans for renal failure, allergies to contrast agents, or financial difficulties and so on. Yang et al. constructed CT radiomics model to evaluate the ability of distinguishing pulmonary granulomatous nodule (GN) from solid lung adenocarcinoma (SADC), and found that plain radiomics (PR) combined with clinical risk factors (PRC) performed better than the other combinations (35). Similarly, Sui et al. extracted radiomics features based on NECT and CECT images, respectively, and confirmed that the radiomics features of plain CT were superior to enhanced CT in predicting the risk of anterior mediastinal lesions (36). By investigating the effect of contrast enhancement, reconstructed layer thickness, and convolution on the discriminative performance of radiomics features of isolated pulmonary nodules, He et al. found that CT based on non-contrast, thin layer, and standard convolution kernels provided more information about the tumor (37). These findings above suggested that the radiomics characteristics of NECT images can detect and describe the biological heterogeneity within the tumor, and the performance of the established model was even better than other phase like CECT images. One underlying reason is that the radiomics information obtained from the non-contrast CT images is not confused by the intravenous contrast material. We believe that with the rapid development of artificial intelligence in the field of medical images, the huge potential value of NECT will be maximized and applied in clinical practice, committing to the realization of personalized and precise treatment of tumors.

With the improvement of image acquisition equipment and imaging quality, the data information generated by images is becoming more and more abundant. However, visual interpretation has obvious limitations and high learning costs as we said before. PET/CT can provide both anatomic and metabolic information, and its advantage in judging distant metastasis of ESCC has been recognized, but its value in assessing lymph node metastasis is still limited with a low sensitivity of 0.64. It is difficult to detect lymph node micro-metastasis and distinguish lymph node metastasis from lymph node reactive hyperplasia or granulomatous inflammation (8). In this study, the AUC of Model 5, which was built by radiomics_NECT_ and radiomics_CECT_ features reached 0.828 and 0.800 in the training and validation dataset, respectively, which means that radiomics was expected to get rid of the bias caused by subjective consciousness between observers and the limitations of traditional diagnostic modes in predicting lymph node metastasis. It reduced learning costs and would be a good assistant to clinical doctors.

There were some limitations in our study. First, this is a retrospective, single-center cohort study, and the reproducibility and generalizability of our results need to be further verified. Previous studies showed that image reconstruction algorithm, preprocessing method, transmission protocol, inter-observer variable and feature extraction algorithm can affect the stability and repeatability of radiomics features (37, 38) How to solve the repeatability problem is the key to future research. Second, we did not incorporate other machine learning methods such as deep learning into our current research, which has been popular in recent years. Third, the region of interest we analyzed was mainly the primary tumor, and information about the area outside the tumor was not obtained, which may also contain some important information. Further studies are warranted to address these questions.

## Conclusion

The model comprised of CT-based radiomics features could accurately predict the lymph node metastasis of esophageal squamous cell carcinoma, and its predictive efficiency was better than the clinical model based on size criteria. Non-contrast-enhanced CT images may contain rich information about tumor heterogeneity, and it could be a reasonable choice for predicting lymph node metastasis of esophageal squamous cell carcinoma.

## Data availability statement

The raw data supporting the conclusions of this article will be made available by the authors, without undue reservation.

## Ethics statement

The studies involving human participants were reviewed and approved by ethics committee of Cancer Hospital of Shantou University Medical College (No.2021121). Written informed consent for participation was not required for this study in accordance with the national legislation and the institutional requirements.

## Author contributions

Conception and design of the study, GP and CC. Acquisition of data, GP, WL, and YW. Analysis and interpretation of the data, GP and CC. All authors participated in clinical data acquisition. Writing and revision of the manuscript, GP and CC. All authors contributed to the article and approved the submitted version.

## Funding

This study was funded by Shantou University Medical College Clinical Research Enhancement Initiative, N0201424 (to CC and JC), Science and Technology Special Fund of Guangdong Province of China, 2019-132 (to CC), Strategic and Special Fund for Science and Technology Innovation of Guangdong Province of China, 180918114960704 (to CC), Innovative Research Group Project of the National Natural Science Foundation of China, 82173079 (to CC).

## Conflict of interest

The authors declare that the research was conducted in the absence of any commercial or financial relationships that could be construed as a potential conflict of interest.

## Publisher’s note

All claims expressed in this article are solely those of the authors and do not necessarily represent those of their affiliated organizations, or those of the publisher, the editors and the reviewers. Any product that may be evaluated in this article, or claim that may be made by its manufacturer, is not guaranteed or endorsed by the publisher.
