# Supplementary material for: Radiomics models based on CT at different phases predicting lymph node metastasis of esophageal squamous cell carcinoma (GASTO-1089)
Source: Front Oncol. 2022 Oct 26;12:988859. doi: 10.3389/fonc.2022.988859 (PMC9643555; doi:10.3389/fonc.2022.988859)
Supplement: Supplementary file 1 [file DataSheet_1.doc]

**Supplementary Table 1. The radiomics features and coefficients of NECT imaging.**

| The radiomics features of NECT | coefficients |
| --- | --- |
| Intercept_ | -9.154125533 |
| original_shape_LeastAxisLength | 0.744477564 |
| original_shape_Elongation | -0.089088088 |
| logarithm_glcm_Correlation | -0.160350446 |
| log.sigma.1.0.mm.3D_firstorder_90Percentile | -0.017084183 |
| log.sigma.1.0.mm.3D_gldm_SmallDependenceLowGrayLevelEmphasis | -0.074539124 |
| log.sigma.2.0.mm.3D_gldm_SmallDependenceLowGrayLevelEmphasis | -0.004246929 |
| wavelet.LHL_firstorder_Skewness | -0.088980291 |
| wavelet.LHH_firstorder_Kurtosis | 0.30835357 |
| wavelet.HLL_glszm_LargeAreaLowGrayLevelEmphasis | 0.124019113 |
| wavelet.HHL_firstorder_Mean | 0.146955551 |
| wavelet.HHL_gldm_SmallDependenceHighGrayLevelEmphasis | -0.122339607 |
| wavelet.HHL_glszm_SizeZoneNonUniformityNormalized | 0.100577896 |
| wavelet.HHL_ngtdm_Strength | -0.063990444 |
| wavelet.HHH_glrlm_RunEntropy | 8.643191643 |
| wavelet.LLL_glszm_LargeAreaLowGrayLevelEmphasis | 0.002457122 |
| logarithm_glcm_Imc1 | 0.16795136 |

Abbreviations: NECT, non-contrast-enhanced CT

**Supplementary Table 2.** **The radiomics features and coefficients of NECT imaging.**

| The radiomics features of CECT | coefficients |
| --- | --- |
| Intercept_ | -7.656740804 |
| original_shape_LeastAxisLength | 0.213589651 |
| original_shape_Elongation | -0.323540197 |
| log.sigma.2.0.mm.3D_glszm_SizeZoneNonUniformityNormalized | 0.239315224 |
| original_ngtdm_Busyness | 0.22365999 |
| log.sigma.0.5.mm.3D_glcm_ClusterShade | -0.004850922 |
| log.sigma.0.5.mm.3D_glszm_SmallAreaEmphasis | 0.946912378 |
| log.sigma.0.5.mm.3D_ngtdm_Busyness | 0.102962564 |
| log.sigma.1.0.mm.3D_firstorder_90Percentile | -0.604484998 |
| log.sigma.1.0.mm.3D_gldm_SmallDependenceLowGrayLevelEmphasis | -0.075983823 |
| log.sigma.1.0.mm.3D_glszm_SmallAreaLowGrayLevelEmphasis | -0.299654584 |
| log.sigma.1.5.mm.3D_gldm_DependenceVariance | 0.010968382 |
| wavelet.LHH_glcm_ClusterShade | 0.030922214 |
| wavelet.HLL_gldm_LargeDependenceEmphasis | 0.54721363 |
| wavelet.HHL_firstorder_Mean | 0.191182238 |
| wavelet.HHL_gldm_LargeDependenceLowGrayLevelEmphasis | 0.335395305 |
| wavelet.HHH_glrlm_RunEntropy | 7.52565967 |
| wavelet.HHH_glszm_SizeZoneNonUniformityNormalized | -0.564078573 |
| square_glrlm_ShortRunLowGrayLevelEmphasis | -0.540548138 |

Abbreviations: CECT,contrast-enhanced CT
